# The potential directing role of chemokines for specific metastatic sites in breast cancer

**DOI:** 10.1038/s41598-026-45036-9

**Published:** 2026-04-09

**Authors:** Alaa M. Ayoub, Motawa E. EL-Houseini, Effat Tharwat, Mohamed F.F Bayomy, Mona S. Abdellateif

**Affiliations:** 1 Misr University for Science and Technology (MUST), 6th of October, Egypt; 2 Medical Biochemistry and Molecular Biology, Cancer Biology Department, National Cancer Institute, Cairo University, Cairo, Egypt; 3 Medical Biochemistry and Molecular Biology, Galala University, Suez City, Egypt

**Keywords:** Cytokines, Chemokines, Breast cancer, Metastasis, Biomarkers, Cancer, Oncology

## Abstract

Metastasis is the most life-threatening sequel in breast cancer (BC). The aim of the study is to assess the association of certain cytokines including IL4, IL-11, CCL-2, CCL-4, and CXCL12 with the site of metastases (lung, bone, brain, ovaries, and liver) in BC. The serum levels of IL4, IL-11, CCL-2, CCL4 and CXCL-12 were assessed in 175 BC patients compared to 50 control subjects using ELISA. The data were correlated to the sites of metastases, patients’ clinicopathological features and response to treatment. The mean age of the BC patients was 49.3 ± 9.5 years old. Distant metastasis was found in 69.1% (121/175) of the patients. There was a significant increase in IL4 (**p = 0.012**), CXCL12 (**p < 0.001**), and CCL4 (**p < 0.001**) in BC patients compared to controls. Lung metastasis associated significantly with increased IL11 concentration (OR = 1.008, *p* = 0.023). Brain metastasis associated with increased IL4 (OR = 1.009, *p* = 0.025), and CXCL12 concentrations (OR = 1.004, *p* = 0.031). Bone metastasis linked with CCL4 (OR = 0.991, *p* = 0.017) and CCL2 (OR = 0.996, *p* = 0.001). Low ER expression, brain, and liver metastasis were considered as potential independent risk factors for shorter disease-free survival (DFS) of BC patients (**p = 0.040**,** 0.001**, and **0.003**; respectively). IL4 (AUC = 0.699, **p = 0.011)** and CXCL12 (AUC = 0.700, **p = 0.010)** showed a diagnostic potential for BC brain metastasis. Combined expression of both IL4 and CXCL12 exhibited a 75% sensitivity and a 71.4% specificity (AUC = 0.768, *p* = 0.001). IL11 associated with the diagnosis of BC lung metastasis (AUC = 0.629, *p* = 0.024). While CCL2 showed a significant potential for the diagnosis of BC liver metastasis (AUC = 0.717, *p* = 0.006). Chemokines have an important role in directing the tumor cells and colonization in a specific metastatic site.

## Background

Breast cancer (BC) is a type of heterogeneous disease comprising different molecular and pathological subtypes. Each subtype has distinct clinical presentation and hormonal susceptibility, that leads to variability in response to therapy, prognosis, and outcomes^1^. It is the commonest malignancy in females and the second cause of cancer-related death in women worldwide^2^. It had been reported that BC was responsible for about 15% of all female deaths in 2022^2^. Though the 5-year survival rate reached up to 99% in locally invasive BC, it rapidly declined to about 30% in case of metastatic set^2^.

Metastasis is a complex process that involves tumor cells spread from its original site to colonize distant parts of the body. It occurs through two distinct pathways; the lymphatic pathway, in which the tumor cells invade the draining lymph nodes through the lymphatic circulation. The other pathway is through blood stream which leads to dissemination of the cells to distant organs^3^. It was found that cancer cells which has the capabilities of spread, invasion, and colonization, are special type of cells with stem-like properties called breast cancer stem cells (BCSCs)^4^. These cells have CD44^+^/CD24^−^/EpCAM^−/+^ phenotype, with a specific molecular profiling (e.g. *PTEN*,* PI3K*,* AKT*,* Wnt*,* β-catenin*,* CD44*,* GJB1 and GDF3*) that figure the tumor behavior and aggressiveness^5,6^. Moreover, these cells were found to produce the chemokine receptor CXCR4, that binds to its ligand chemokine CXCL-12, which is largely expressed in lymph nodes, liver, bone, and lung^7^. Therefore, the BC cells are preferentially metastasized to the lung, bones, and liver^3^.

There are many theories described the homing of tumor cells in a destination organ, among them are the “Seed and soil” hypothesis that proposed by Stephen Paget^8^. Accordingly, the migration of cancer cells is influenced by the Pre-metastatic niche, which describes the altered tissue environment of the secondary organ to facilitate metastatic growth in response to certain factors produced by the primary cancer^9,10^. Therefore, various organs have remarkable abilities to stop or attract certain types of cancer cells through different substances known as chemotactic factors^11^. These chemotactic factors included some growth factors, cytokines, and chemokines which induce vascular permeability and recruitment of suppressive immune cells in the extracellular matrix (ECM) and the secondary organs^9,12^. The formed Pre-metastatic niche can facilitate the attraction and extravasation of circulating tumor cells (CTCs) to the destination organs and provides a favorable environment for the proliferation of incoming cancer cells^10^. For instance, lung epithelial cells release chemokines when activated by tumor-derived RNAs, which results in the recruitment of bone marrow derived cells (BMDCs) and CTCs to facilitate the adhesion of BC cells to the lung tissue^13^.

Chemokines are a distinct family of cytokines known as “chemoattractant cytokines,” which are essential for the directed cellular migration and movement^14^. Numerous receptors for chemokines and cytokines can influence the host-tumor interactions and enable cancer cells to escape the immune response by attracting immunoregulatory cells such as T-regulatory cells and tumor-associated macrophages (TAMs), thereby creating an immunosuppressive tumor microenvironment^15^. In another words, chemokines influence tumor behavior and outcome through various mechanisms. One of these mechanisms involves chemokines facilitating angiogenesis, which aids in tumor growth and metastasis. Another mechanism is the recruitment of either promoting or inhibiting leukocytes. Lastly, they can function as growth factors for tumor cells, potentially contributing to either tumor progression or regression^16^. Some series classified cytokines in BC tumor microenvironment (TME) into two groups: protumor cytokines such as CCL2, CCL5, CCL18, CCL3, CCL22, CXCL8, and CXCL12. While the other antitumor cytokines group included CXCL9, CXCL10, CXCL4, IL2, IFN-γ, IL15 and CXCL11^17,18^.

Accordingly, the assigned cytokines were selected based on their important function in immune modulation, inflammation, and chemokine-mediated cell recruitment. IL4 and IL11 represent anti-inflammatory and regulatory cytokines, while CCL2, CCL4, and CXCL12 are confirmed as key regulators in leukocyte trafficking, cellular migration, and metastasis. Hence, the combined assessment of these cytokines allows evaluation of TME, pre-metastatic niche, inflammatory and regulatory immune responses relevant to the study objectives.

Therefore, the aim of the present study is to assess the association of certain cytokines including IL4, IL-11, CCL-2, CCL-4, and CXCL12 with the site of metastases (lung, bone, brain, ovaries, and liver) in BC patients. Additionally, to evaluate the impact of the serum levels of IL4, IL-11, CCL-2, CCL-4 and CXCL12 on the patients’ clinicopathological features and response to treatment.

## Methods

The present study included 175 BC patients compared to 50 healthy age and sex matched control subjects. Patients were presented and diagnosed at the National Cancer Institute (NCI), Cairo University. Female control subjects were those presented to the NCI for blood donation, aged (20–50 years old). They were confirmed with laboratory investigations as viral free, and had no chronic diseases.

Participants included in the study were those aged 20–75 years old. Also, those who confirmed pathologically with breast carcinoma, whether metastatic, non-metastatic, or locally advanced. The non-eligible patients for involvement in the study were those with a history of prior malignancy, previous receipt of chemotherapy in either adjuvant or metastatic settings, significant uncontrolled comorbidities, and insufficient organ function that would contraindicate the use of any of the prescribed treatments. Patients underwent a comprehensive assessment that included detailed medical history, clinical evaluation, and imaging studies such as plain chest X-rays, abdominal ultrasounds, and bone scans with CT scans performed if necessary. Follow-up evaluations were conducted after three and six cycles of therapy. Assessment of Patient’s response to therapy was performed according to the Response Evaluation Criteria in Solid Tumors (RECIST) criteria^19^.

The concentration levels of IL4, IL-11, CCL-2, CCL4 and CXCL-12 were assessed in patients with metastatic breast cancer in comparison to non-metastatic breast cancer patients and normal control group. The high and low levels of cytokines in cancer patients were defined according to the median value.

Sample size Calculation was performed using online power test for sample size calculation, taking into consideration the incidence of primary outcome in population and study group with an α-error of 0.05 and power of 80%.

The research protocol was approved by the NCI ethical committee which was in concordance with the 2011 declaration of Helsinki [no. 2307-302-076]. All participated subjects signed informed consent for involvement in the study.

### Sample collection

Peripheral blood samples (5 ml) were drained from patients and control subjects in serum separator tubes during their follow-up visits. The samples were left to clot for 10 to 20 min at room temperature, and then they were centrifuged at 1000 g for 10 min. After that the serum was aliquot and stored at −80 °C until use for evaluation of the protein level of IL4, IL11, CCL4, CCl2, and CXCL12.

### Assessment of the cytokines levels

The serum levels of IL4, IL11, CCL4, CCl2, and CXCL12 were assessed in the participating BC patients and the control groups using Enzyme-Linked Immunosorbent Assay (ELISA) technique. The steps for measuring the concentrations of the assessed markers were performed according to the supplied manufactures’ instructions (Shanghai YL Biotech Co., Ltd, Zhangjiang Town) as follows: IL4 (Cat. No. YLA1519HU), IL11 (Cat. No. YLA1524HU), CCL4 (Cat. No. YLA3757HU), CCl2 (Cat. No. YLA4287HU), and CXCL12 (Cat. No. YLA1067HU).

### Statistical analysis

The SPSS software (version 22; SPSS Inc., Chicago, IL, USA) was used to analyze the data. The Shapiro test was used to test for normalization of the values. Cytokine concentrations were analyzed in their original units without normalization or log transformation. Nonparametric statistical tests were applied as the data did not meet assumptions of normality. The Mann-Whitney or Kruskal-Wallis tests were used to compare groups, where continuous variables were expressed as median and interquartile range (IQR). The Chi-square test was used to compare groups when categorical variables were displayed as frequencies and percentages. Bonferroni test was used for validation. The diagnostic utility of the assessed markers for metastasis in BC patients was evaluated using a receiver operating characteristic (ROC) curve analysis. To find the relationship between various variables and the metastatic sites in BC patients, univariate and multivariate regression analyses were conducted. The date of primary treatment was used to calculate disease-free survival (DFS). Cox-regression hazard model was used to assess factors associated with disease recurrence. Statistical significance was considered when *p* < 0.05, where all the conducted tests were two-tailed.

## Results

### Clinical features of the assessed BC patients

The mean age of the included BC patients was 49.3 ± 9.5 years old, with a median of 50 (range: 20–75) years. Most of the patients were diagnosed with intra-ductal carcinoma 90.3% (158/175) and 8.6%1(5/175) had intralobular carcinoma. 74.9% (131/175) of the patients had grade 2, 24% (42/175) were grade 3, and only 2 (1.1%) patients were grade 1. There were 21/175 (12%) patients with TNBC, and LN affection was shown in 86.3% (151/175) of the patients. Metastasis to distant organs was found in 69.1% (121/175) of the patients, where 35 (28.9%) metastasize to the bone, 23 (19.0%) to the lung, 16 (13.2%) to the liver, 8 (6.6%) to the brain, 17 (14%) to both liver and bone, and 10 (8.3%) to the lung and bone. There were 86.3% (151/175) of the patients showed recurrent disease, and only 30.9% (54/175) were responsive to therapy (Table 1).

**Table 1:** Clinical features of the assessed BC patients.

Test variables		Frequency (%)
Age (years)	mean ± SD	49.3 ± 9.5
	Median (IQR)	50 (20–75)
tumor grade	1	2 (1.1%)
	2	131 (74.9%)
	3	42 (24%)
tumor type	IDC	158 (90.3%)
	ILC	15 (8.6%)
	IDC&ILC	2 (1.1%)
Hormonal status	NTNBC	154 (88%)
	TNBC	21 (12%)
ER expression	negative	55 (31.4%)
	positive	120 (68.6%)
PR expression	negative	41 (23.4%)
	positive	134 (76.6%)
HER2 expression	negative	100 (57.1%)
	positive	75 (42.9%)
LNs affection	negative	24 (13.7%)
	positive	151 (86.3%)
Metastasis	negative	54 (30.9%)
	positive	121 (69.1%)
Site of metastasis	bone	35 (28.9%)
	lung	23 (19.0%)
	liver	16 (13.2%)
	liver&bone	17 (14.0)
	lung&bone	10 (8.3%)
	brain	8 (6.6)
	bone&brain	4 (3.3%)
	liver, bone&brain	4 (3.3%)
	liver, lung&bone	4 (3.3%)
Response to treatment	responsive	54 (30.9%)
	nonresponsive	121 (69.1%)
Recurrence	negative	24 (13.7%)
	positive	151 (86.3%)

### Expression profile of the assessed markers in BC patients

**Fig. 1:**
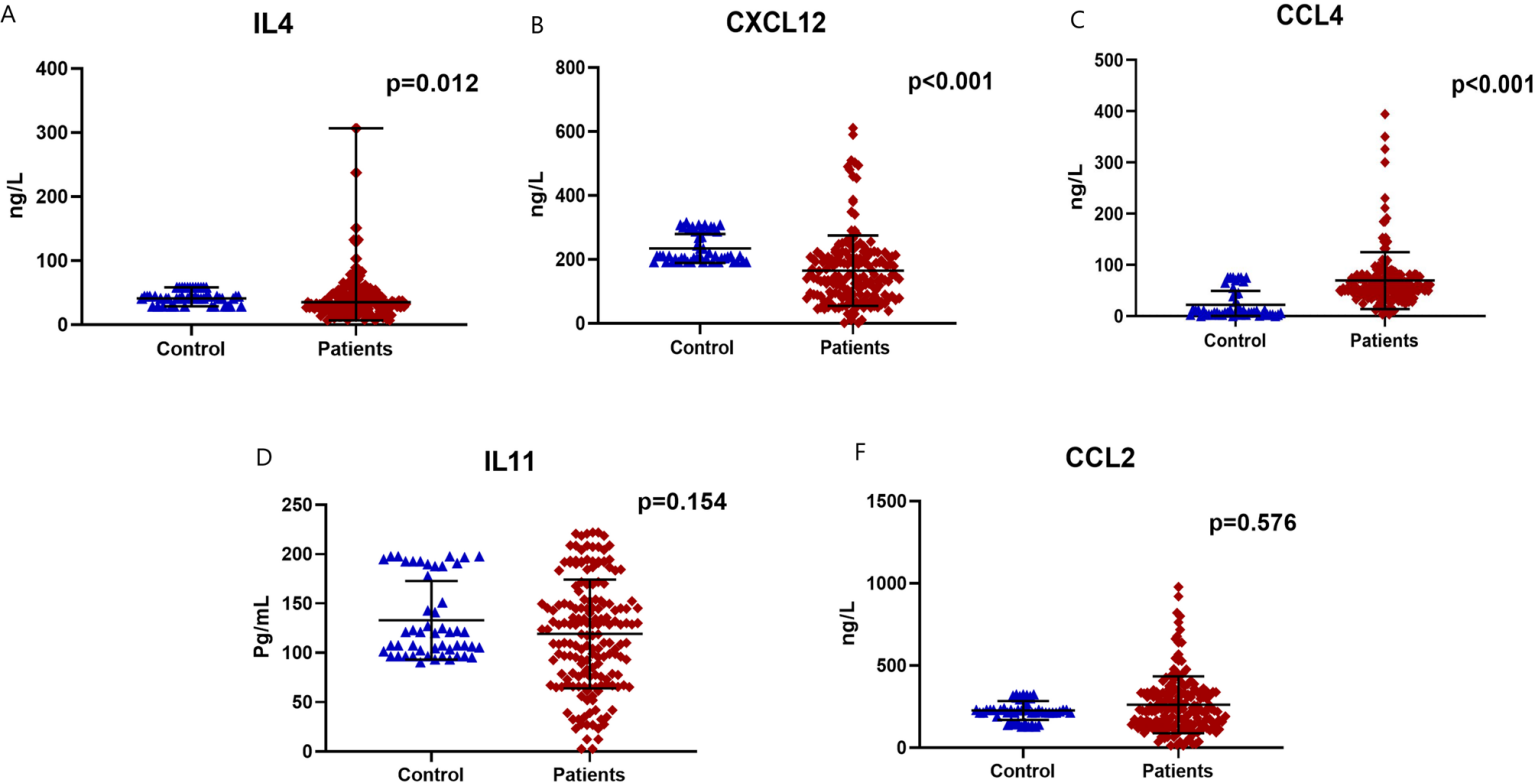
The expression profile of the assessed markers **A**) IL4, **B**) CXCL12, **C**) CCL4, **D**) IL11, and **E**) CCL2 in BC patients compared to normal controls.

There was a significant increase in IL4 level in BC patients compared to the control females [35.3 (6.7–307) versus 40.97(28.7–58.5) ng/L; respectively, *p* = 0.012]. Also, CXCL12 was significantly increased in BC patients compared to the control group [142.2 (1.9–611) and 210.9 (192–308.5.5) ng/L; respectively, *p* < 0.001]. Similarly, CCL4 was substantially elevated in BC females in comparison to the control subjects [55.5 (0.1–394) versus 6.8 (0.1–75.2) ng/L; respectively, *p* < 0.001]. On the other hand, there was no significant difference between the BC patients and the control females regarding IL11 [123.6 (2.5–221.9.5.9) and 121.1 (96.5–197.9.5.9) pg/mL; respectively, *p* = 0.154], and CCL2 concentrations [230.8 (12.9- 977.8) and 231.2 (129–324) ng/L; respectively, *p* = 0.576], **(**Fig. 1**)**.

### Association of IL4, IL11, CCL4, CCl2, and CXCL12 with the clinic-pathological features of the BC patients

There was a significant increase in IL4 level in BC patients who had brain metastasis [42 (21.3–306.9.3.9) ng/L], compared to those without brain metastasis [31.1 (7.4–237.4.4.4) ng/L, (**p = 0.011**)]. Also, IL4 was greatly increased in patients with disease recurrence [33.3 (6.7–306.9.7.9) ng/L] in relation to those with no recurred disease [46.8 (24.7–78.6) ng/L, (**p = 0.009**)]. Additionally, there was a notable increase of IL11 in non-TNBC patients [128 (IQR: 2.5–221.9.5.9)], compared to the TNBC group [107 (IQR: 27–152.6.6), *p* = 0.036]. Moreover, patients with positive estrogen receptors (ER) showed significantly increased levels of IL11 (**p = 0.027**) and CCL4 (**p = 0.018**) [129.5 (IQR: 2.5–222) pg/mL and 58.2 (IQR: 3.2–394) ng/L; respectively], compared to BC patients with low/negative ER expression [117.5 (IQR: 27–204) pg/mL, and 51.1 (IQR: 1–356.9.9) ng/L; respectively]. IL11 was also significantly increased in BC patients with lung metastasis in relation to those who had no lung metastasis [110 (IQR: 32.5–221.9.5.9) versus 105.8 (IQR: 23–207) pg/mL; respectively, *p* = 0.012]. Furthermore, the increased CCl2 level associated significantly with distant metastasis and poor response to treatment (**p = 0.001** for both). Regarding the CXCL12 expression, there was a substantial decrease in CXCL12 level in ER positive patients [139 (IQR: 1.9–411) ng/L] compared to those with ER negative group [200 (IQR: 11–502) ng/L, *P* = 0.006]. Additionally, it was significantly associated with HER2 expression and with distant metastasis (**P < 0.001** and *p* = 0.001; respectively). The CXCL12 was also increased significantly in patients with brain metastasis [118.8 (1.9–502)] ng/L, in relation to those who had no brain metastasis [208 (88.8–611.0) ng/L, *p* = 0.010]. Recurrence of BC was significantly experienced in patients with increased level of CXCL12 [179.7 (124–251) ng/L] compared to those with low CXCL12 concentration [139 (1.9–611.2.9.2) ng/L, *p* = 0.041]. Moreover, BC patients with elevated CXCL12 concentration showed poor response to therapy in comparison to those who had low level of CXCL12 [179.9 (29.6–266), versus 121.8 (1.9–611) ng/L; respectively, *p* = 0.001, Tables 2 and 3**].**

**Table 2:** Association of IL4, CXCL12, and CCL4 with the clinico-pathological features of the BC patients.

		IL4 (ng/L)		CXCL12 (ng/L)	P value	CCL4 (ng/L)	P value
Tumor grade	1	8.4 (8–8.9.9)	0.130	140.6 (140–141)	0.623	109 (109–109.6.6)	**0.022**
	2	35.6 (6.7–307)		142 (1.9–411)		58.6 (1–394)	
	3	34.4 (7.4–237)		146.9 (39–381)		52.6 (1–356.9.9)	
Tumor type	IDC	35.6 (6.7–306.9.7.9)	0.525	150 (11–411.2.2)	0.068	57.8 (1–394.3.3)	0.626
	ILC	25 (7.4–89.3)		98.9 (11–591.2.2)		54.9 (24–191)	
	IDC&ILC	29 (28.8–30)		179.8(178–180)		51 (49.2–53)	
Hormonal status	non-TNBC	35.9 (6.7–237.4.7.4)	0.582	150 (1.9–411)	0.272	57.8 (1–394.3.3)	0.156
	TNBC	28.2 (18.2–306.9.2.9)		98.9 (11–502.8.8)		44.8 (1–101.9.9)	
LNs affection	negative	36.4 (8.9–65.7)	0.722	149.7 (69–387)	0.057	67.5 (23.8–110)	0.344
	positive	35.2 (6.7–306.9.7.9)		135.8 (1.9–411)		55 (1–394.3.3)	
ER expression	negative	39 (17–306.9.9)	0.051	200 (11–502)	**0.006**	51.1 (1–356.9.9)	**0.018**
	positive	33.4 (6.7–151)		139 (1.9–411)		58.2 (3.2–394)	
PR expression	negative	36.8 (12–306.9.9)	0.181	146.9 (11–502.8.8)	0.506	51.1 (1–356.9.9)	0.319
	positive	35.2 (6.7–129.5.7.5)		141.7 (1.9–411)		58.6 (1–394)	
HER-2 expression	negative	31.7 (6.7–306.9.7.9)	0.297	133.2 (1.9–503)	*P* < 0.001	58.2 (1–394.3.3)	0.569
	positive	36.8 (10–237)		191.8 (48.4–611)		52.3 (1–356.9.9)	
Distant Metastasis	negative	38.5 (6.7–129.5.7.5)	0.083	179.7 (29.6–266)	**0.001**	59.9 (1–109.6.6)	0.868
	positive	33.3 (7.4–306.9.4.9)		121.8 (1.9–611)		52.6 (11–394.3.3)	
Bone (74/121)	negative	36 (10–306.9.9)	0.404	146.9 (50.6–411)	0.221	58.6 (1–394.3.3)	0.102
	positive	31.4 (7.4–132.8.4.8)		92 (1.9–502.8.9.8)		49.7 (11–190.9.9)	
Liver (41/121)	negative	32.6 (12.4–306.9.4.9)	0.324	135.8 (11–411)	0.875	53.8 (1–190.9.9)	0.153
	positive	35.2 (7.4–237.4.4.4)		143 (50–481)		49 (11–394.3.3)	
Lung (37/121)	negative	31.7 (7.4–306.9.4.9)	0.083	116.7 (1.9–411)	0.770	50 (1–394.3.3)	0.237
	positive	36.8 (13–151)		131.6 (45–386.6.6)		58.6 (3.2–190.9.2.9)	
Brain (16/121)	negative	31.1 (7.4–237.4.4.4)	**0.011**	118.8 (1.9–502)	**0.010**	52.6 (3.2–394.3.2.3)	0.178
	positive	42 (21.3–306.9.3.9)		208 (88.8–611.0)		49.7 (1–101.9.9)	
Response to treatment	responsive	39 (6.7–129.5.7.5)	0.085	179.9 (29.6–266)	**0.001**	60.9 (1–109.6.6)	0.617
	Non-responsive	33.3 (7.4–306.9.4.9)		121.8 (1.9–611)		52.3 (1–394.3.3)	
Recurrence	negative	46.8 (24.7–78.6)	**0.009**	179.7 (124–251)	**0.041**	53.5 (1–97.1.1)	0.162
	positive	33.3 (6.7–306.9.7.9)		139 (1.9–611.2.9.2)		55.5 (1–394.3.3)	

**Table 3:** Association of IL11, CCl2, and with the clinic-pathological features of the BC patients.

Test variables		IL11 (pg/mL)	*P* value	CCL2 (ng/L)	*P* value
tumor grade	1	142.5 (140 − 45)	0.843	455.5(455–456)	0.078
	2	123.6 (2.5–222)		235.5 (13–890)	
	3	103.6 (12.4–221)		222.5 (36.9–977.8.9.8)	
tumor type	IDC	114 (2.5–222)	0.272	230.6 (12.9–977.8.9.8)	0.124
	ILC	144.7 (65.4–121)		175.5 (65.6–456)	
	IDC&ILC	168.8 (167.6–170)		477.5 (476.9–478)	
Hormonal status	NTNBC	128 (2.5–221.9.5.9)	**0.036**	230.8 (12.9–977.8.9.8)	0.960
	TNBC	107 (27–152.6.6)		297.8 (22.5–889.7.5.7)	
LNs affection	negative	142.4 (39–171.8.8)	0.080	249 (12.9–456)	0.561
	positive	110 (2.5–221.9.5.9)		225 (18.1–977.8.1.8)	
ER expression	negative	117.5 (27–204)	**0.027**	231.6 (22.5–977.8.5.8)	0.504
	positive	129.5 (2.5–222)		223 (12.9–763.6.9.6)	
PR expression	negative	117.5 (27–220.7.7)	0.167	236.4 (12.9–977.8.9.8)	0.614
	positive	128.4 (2.5–221.9.5.9)		230.8 (18.1–717.6.1.6)	
HER-2 expression	negative	123 (2.5–209)	0.466	239 (12.9–889.7.9.7)	0.693
	positive	123.6 (32.5–222)		227.7 (58.5–977.8.5.8)	
Distant Metastasis	negative	134 (2.5–218.6.5.6)	0.195	308 (90.5–544.7.5.7)	**0.001**
	positive	108.7 (23–221.9.9)		171.7 (12.9–977.8.9.8)	
Bone metastasis (74/121)	negative	109 (27–221)	0.577	243 (35–977.8.8)	0.071
	positive	104 (23–221.9.9)		165.6 (12.9–436.7.9.7)	
Liver metastasis (41/121)	negative	120.6 (23–221.9.9)	0.019	175.5 (18–889.7.7)	0.961
	positive	77.6 (27.7–204.2.7.2)		168.6 (12.9–977.8.9.8)	
Lung metastasis (37/121)	negative	105.8 (23–207)	**0.012**	168.6 (12.9–977.8.9.8)	0.813
	positive	110 (32.5–221.9.5.9)		175.5 (18–763.6.6)	
Brain metastasis (16/121)	negative	109 (23.2–221.9.2.9)	0.335	171.7 (12.9–978)	0.095
	positive	103 (27–207.4.4)		262.7 (108–889.7.7)	
Response to treatment	responsive	134 (2.5–218.6.5.6)	0.195	308 (90.5–544.7.5.7)	**0.001**
	nonresponsive	108.7 (23–352.4.4)		175.5 (13–977.8.8)	
Recurrence	negative	123.9 (56–218.6.6)	0.938	326.5 (90.5–544.7.5.7)	0.068
	positive	123.6 (2.5–352.4.5.4)		222.7 (12.9–977.8.9.8)	

### Association of IL4, IL11, CCL4, CCL2, and CXCL12 with the metastatic site in BC patients

**Fig. 2:**
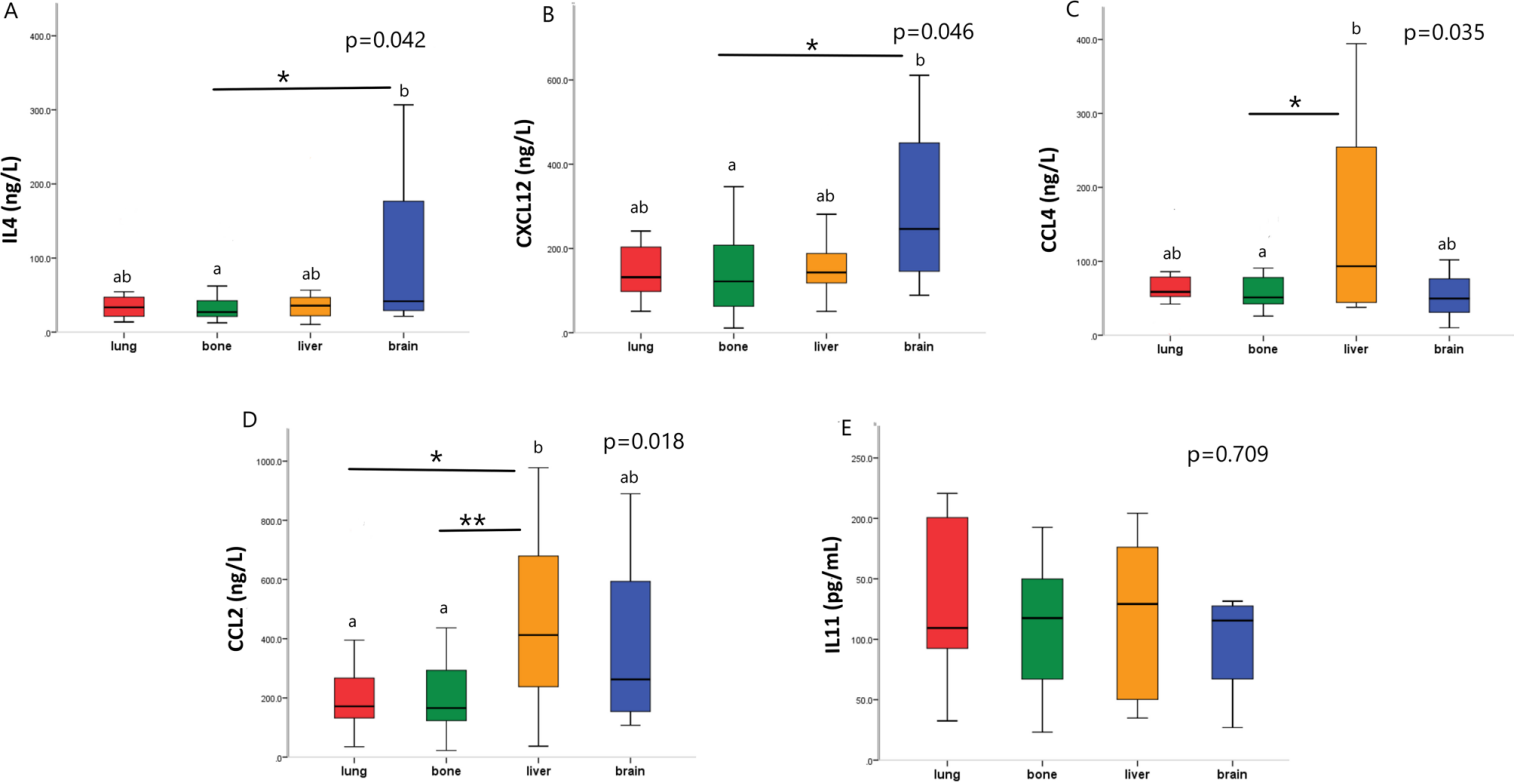
Association of **A**) IL4, **B**) CXCL12, **C**) CCL4, **D**) CCL2, and **E**) IL11 with the metastatic site in BC patients. *p < 0.05, **p < 0.005.

Kruskal Wallis test with multiple comparisons were performed to assess the association of IL4, IL11, CCL4, CCL2, and CXCL12 with each metastatic site in BC patients. Increased IL4 level was significantly associated with brain metastasis [41.6 (21–306.9.9) ng/L] in comparison to bone metastasis [27 (12.4–32.8) ng/L, p = **0.042**]. However, there was no significant difference in IL4 levels regarding liver, lung, and brain metastasis (Fig. 2A). Increased CXCL12 level associated substantially with brain metastasis [246 (88.8–611) ng/L] in comparison to bone metastasis [121.8 (11–502.8.8) ng/L, *p* = 0.046]. While there were no significant differences among liver, lung, and brain metastasis regarding CXCL12 concentration (Fig. 2B). The CCL4 concentration was significantly increased in patients with liver metastasis [93 (37.8–394) ng/L] in comparison to those with bone metastasis [51 (25.8–145) ng/L, *p* = 0.035]. While there was no significant difference among lung, brain, and liver metastasis (Fig. 2C). Similarly, the increased CCL2 level associated significantly with liver metastasis [412.6 (37–977.8.8) ng/L] in relation to bone [166 (22.5–436.7.5.7) ng/L, *p* = 0.004] or lung metastasis [171.7 (35–763.6.6) ng/L, *p* = 0.009]. However, there was no significant difference between liver and brain metastasis regarding CCL2 concentration. Also, there was no significant difference in CCL2 levels among bone, lung, and brain metastasis (Fig. 2D). On the other hand, IL11 level did not show any significant association with bone, lung, liver, or brain metastasis (*p* = 709, Fig. 2E; Table 4).

**Table 4:** Association of IL4, IL11, CCL4, CCL2, and CXCL12 with the metastatic site in BC patients.

	Bone	Lung	liver	brain	*P* value
**IL4 **(ng/L)	27 (12.4–32.8) ^a^	33.5 (13.7–151) ^ab^	35.6 (10–237.4.4) ^ab^	41.6 (21–306.9.9) ^b^	**0.042**
**CXCL12** (ng/L)	121.8 (11–502.8.8) ^a^	131.6 (51–386.6.6) ^ab^	143 (50.6–481) ^ab^	246 (88.8–611) ^b^	**0.046**
**CCL4** (ng/L)	51 (25.8–145) ^a^	58.6 (3.2–184) ^ab^	93 (37.8–394) ^b^	49.7 (10–102) ^ab^	**0.035**
**CCL2** (ng/L)	166 (22.5–436.7.5.7) ^a^	171.7 (35–763.6.6) ^a^	412.6 (37–977.8.8) ^b^	262.7 (108–889.7.7) ^ab^	**0.018**
**IL11** (pg/mL)	117.5 (23–192.6.6)	109 (32.5–220.7.5.7)	129 (34.8–221)	115.5 (27–131.5.5)	0.709

### Univariate and Multivariate regression analysis for predictors of metastatic site in breast cancer

Univariate analysis showed that lung metastasis associated significantly with IL11 concentration (OR = 1.008, *p* = 0.023). Brain metastasis in BC patients associated significantly with increased IL4 concentration (OR = 1.009, *p* = 0.025), CXCL12 concentration (OR = 1.004, *p* = 0.031), negative ER expression (OR = 0.150, *p* = 0.001), negative PR expression (OR = 0.313, *p* = 0.034), and increased HER2 expression (OR = 3.063, *p* = 0.044). Bone metastasis linked significantly with CCL4 (OR = 0.991, *p* = 0.017), CCL2 (OR = 0.996, *p* = 0.001), and LNs affection (OR = 5.268, *p* = 0.048). Finally, liver metastasis associated substantially with LNs involvement (OR = 6.686, *p* = 0.024), and HER2 expression (OR = 2.547, *p* = 0.018).

The multivariate analysis revealed that IL4 (OR = 1.010, *p* = 0.016), CXCL12 (OR = 1.004, *p* = 0.023), and ER (OR = 0.195, *p* = 0.035) expressions are independent risk factors for brain metastasis in BC patients. While CCL2 expression (OR = 0.996, *p* = 0.006), and LNs affection (OR = 10.374, *p* = 0.008) are independent risk factors for bone metastasis in BC patients. Moreover, LNs involvement (OR = 6.551, *p* = 0.029), and HER2 expression (OR = 2.519, *p* = 0.023) are considered independent risk factors for liver metastasis in BC patients (Table 5).

**Table 5:** Univariate and Multivariate regression analysis for predictors of metastatic site in breast cancer.

	Univariate regression analysis				Multivariate regression analysis			
	OR	95% C.I.		P value	OR	95% C.I.		P value
		Lower	Upper			Lower	Upper	
**Lung metastasis**								
IL4	1.002	0.994	1.009	0.639				
IL11	1.008	1.001	1.015	**0.023**				
CXCL12	0.999	0.995	1.002	0.372				
CCL4	1.000	0.994	1.006	0.966				
CCL2	0.999	0.997	1.001	0.420				
TNBC	0.000	0.000	.	0.998				
LNs	1.346	0.259	7.004	0.724				
ER	1127	0.000	.	0.998				
PR	4.350	1.404	13.479	0.011				
HER2	1.255	0.562	2.803	0.579				
**Brain metastasis**								
IL4	1.009	1.001	1.017	**0.025**	1.010	1.002	1.018	**0.016**
IL11	0.995	0.985	1.004	0.279				
CXCL12	1.004	1.000	1.007	**0.031**	1.004	1.001	1.008	**0.023**
CCL4	0.987	0.970	1.005	0.149				
CCL2	1.001	0.999	1.004	0.198				
ER	0.150	0.049	0.459	**0.001**	0.195	0.042	0.895	**0.035**
PR	0.313	0.106	0.918	**0.034**	0.987	0.192	5.076	0.988
HER	3.063	1.031	9.096	**0.044**	3.166	0.892	11.238	0.075
LNs	266	0.000	.	0.999				
TNBC	2.000	0.569	7.028	0.280				
**Bone metastasis**								
IL4	0.992	0.984	1.001	0.069				
IL11	0.997	0.991	1.003	0.395				
CXCL12	0.997	0.995	1.000	0.086				
CCL4	0.991	0.984	0.998	**0.017**	0.997	0.988	1.006	0.542
CCL2	0.996	0.994	0.999	**0.001**	0.996	0.993	0.999	**0.006**
LNs	5.268	1.016	27.312	**0.048**	10.374	1.862	57.809	**0.008**
ER	0.992	0.429	2.294	0.986				
PR	1.228	0.544	2.772	0.621				
HER2	0.501	0.236	1.061	0.071				
**Liver metastasis**								
IL4	0.998	0.989	1.006	0.561				
IL11	0.994	0.988	1.001	0.083				
CXCL12	0.997	0.994	1.001	0.099				
CCL4	1.003	0.998	1.009	0.226				
CCL2	1.001	0.999	1.003	0.299				
TNBC	0.468	0.145	1.516	0.206				
LNs	6.686	1.285	34.786	**0.024**	6.551	1.218	35.237	**0.029**
ER	0.520	0.225	1.204	0.127				
PR	0.860	0.372	1.987	0.724				
HER2	2.547	1.174	5.528	**0.018**	2.519	1.137	5.581	**0.023**

### Diagnostic significance of IL4, IL11, CCL4, CCL2, and CXCL12 in relation to the metastatic site in breast cancer patients

Roc curve analysis was performed to assess the diagnostic potential of the examined cytokines with the metastatic sites in BC patients. The data revealed that IL4 had a sensitivity of 75% and a specificity of 61.9%, at a cutoff value of 36.4 ng/L (AUC = 0.699, *p* = 0.011) for the association with brain metastasis. Similarly, CXCL12 achieved a sensitivity of 62.5% and a specificity of 81%, at a cutoff value of 197.9 ng/L (AUC = 0.700, *p* = 0.010) for the association with brain metastasis in BC. While the combined expression of both IL4 and CXCL12 exhibited a 75% sensitivity and a 71.4% specificity (AUC = 0.768, *p* = 0.001) for brain metastasis in BC patients.

**Fig. 3:**
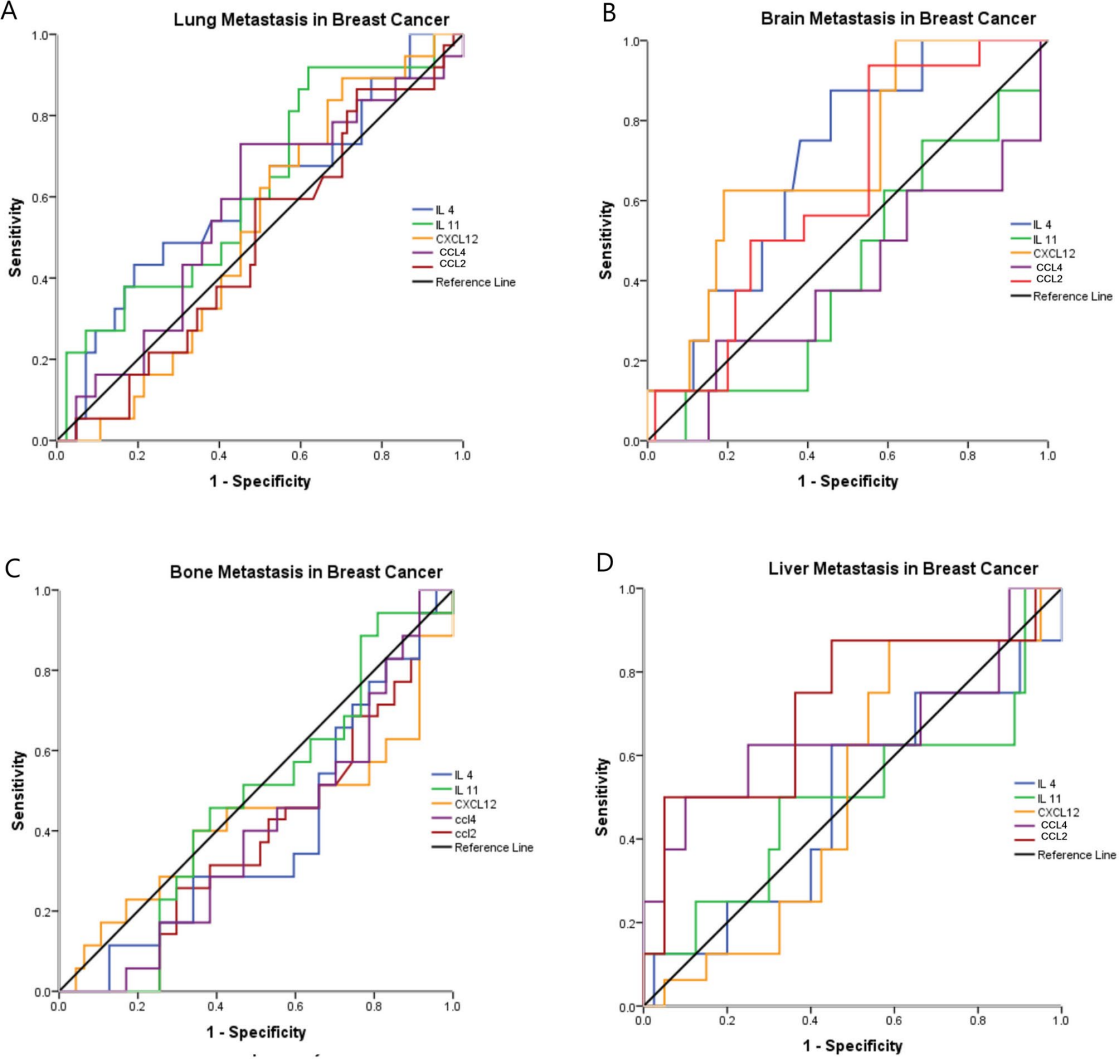
Assessment of the diagnostic significance of IL4, IL11, CCL4, CCL2, and CXCL12 in relation to the metastatic site **A**) Lung, **B**) Brain, **C**) Bone, and **D**) Liver in breast cancer patients.

Regarding lung metastasis, IL11 showed a sensitivity of 81.1%, and a specificity of 42.9% at a cutoff value of 88.8 pg/mL (AUC = 0.629, *p* = 0.024) for the diagnosis of lung metastasis in BC patients. Moreover, CCL2 level showed a significant potential for the diagnosis of liver metastasis in BC patients with a sensitivity, specificity, and AUC of (75%, 63.7%, and 0.717; respectively, *p* = 0.006), at a cutoff value of 248 ng/L (Fig. 3).

On the other hand, the plasma levels of IL4, IL11, CCL4, CCL2, and CXCL12 did not show any significant potential for bone metastasis in BC patients. Other data were illustrated in Table 6.

**Table 6:** Diagnostic significance of IL4, IL11, CCL4, CCL2, and CXCL12 for the metastatic site in breast cancer patients.

Variables	Brain Metastasis					Lung Metastasis				
	AUC	cutoff	sensitivity	specificity	*P* value	AUC	cutoff	sensitivity	specificity	*P* value
**IL 4**	0.699	36.4	75%	61.9%	**0.011**	0.529	47.9	43.2%	81%	0.083
**IL 11**	0.423	78.2	62.5%	41%	0.320	0.629	88.8	81.1%	42.9%	**0.024**
**CXCL12**	0.700	197.9	62.5%	81%	**0.010**	0.517	113.4	62.2%	50%	0.770
**CCL4**	0.398	48.4	62.5%	35.2%	0.188	0.566	51.6	73%	55%	0.247
**CCL2**	0.630	153.6	93.8%	44.8%	0.095	0.486	170	59.5%	51.2%	0.813
**IL4 + CXCL12**	0.768	-	75	71.4	**0.001**					
Variables	**Liver Metastasis**					**Bone Metastasis**				
	AUC	cutoff	sensitivity	specificity	P value	AUC	cutoff	sensitivity	specificity	P value
**IL 4**	0.491	34.4	62.5%	55%	0.906	0.391	26.2	54.3%	33%	0.094
**IL 11**	0.495	108	62.5%	42.5%	0.953	0.469	113.4	51.4%	53.2%	0.629
**CXCL12**	0.512	137.4	62.5%	51.2%	0.875	0.421	118.7	51.4%	33%	0.221
**CCL4**	0.652	80.5	62.5%	75%	0.056	0.394	50.7	51.4%	33%	0.102
**CCL2**	0.717	248	75%	63.7%	**0.006**	0.383	159.9	51.4%	33%	0.071

### Disease Free Survival of the patients

**Fig. 4:**
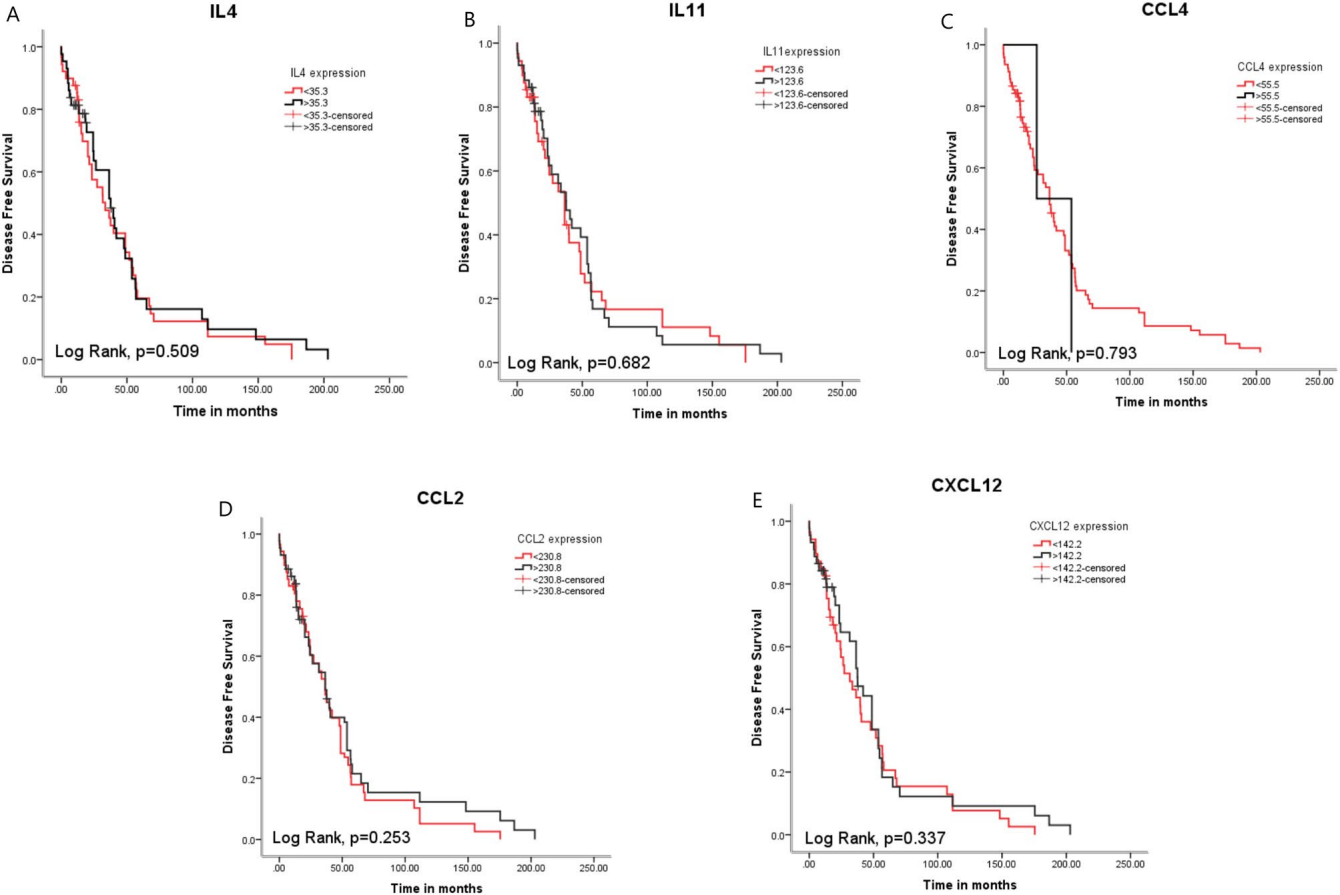
Assessment of the impact of **A**) IL4, **B**) IL11, **C**) CCL4, **D**) CCL2, and **E**) CXCL12 on the DFS rates of the BC patients.

The mean DFS time of the included patients was 47.602 months. There was no significant impact of IL4, IL11, CCL4, CCL2, and CXCL12 on the DSF rate of the assessed BC patients (*p* = 0.509, 0.682, 0.793, 0.253, and 0.337; respectively, Fig. 4).

Cox regression hazard model was performed to assess the factors associated with disease recurrence of the patients. It showed that LNs affection [HR = 1.805, *p* = 0.013], ER expression [HR = 1.870, *p* = 0.001], metastasis to the lung [HR = 1.766, *p* = 0.005], brain [HR = 2.448, *p* = 0.001], and liver [HR = 1.855, *p* = 0.002] associated significantly with shorter DFS of the patients. Additionally, low ER expression, brain, and liver metastasis were independent risk factors for shorter DSF rate of the BC patients (**p = 0.040**,** 0.001**, and **0.003**; respectively, Table 7).

**Table 7:** Cox regression analysis for disease-free survival (DFS) in BC patients.

**Test variable**	**Univariate Analysis**				**Multivariate Analysis**			
	**HR**	**95.0% CI **		**P value**	**HR**	**95.0% CI **		**P value**
		**Lower**	**Upper**			**Lower**	**Upper**	
LNs	1.805	1.133	2.876	**0.013**	1.242	0.563	2.738	0.592
Metastasis	1.374	0.904	2.087	0.137				
TNBC	1.376	0.847	2.237	0.198				
ER	1.870	1.273	2.749	**0.001**	1.660	1.025	2.689	**0.040**
PR	1.293	0.874	1.913	0.199				
HER	1.032	0.740	1.439	0.852				
lung	1.766	1.186	2.630	**0.005**	1.333	0.854	2.080	0.206
brain	2.448	1.426	4.202	**0.001**	0.376	0.210	0.671	**0.001**
liver	1.855	1.259	2.732	**0.002**	0.531	0.349	0.808	**0.003**
bone	0.698	0.477	1.023	0.065				
IL4	1.113	0.803	1.543	0.521				
IL11	1.068	0.772	1.480	0.690				
CCL4	0.877	0.323	2.382	0.797				
CCL2	1.203	0.868	1.668	0.267				
CXCL12	1.168	0.842	1.621	0.351				

## Discussion

Metastasis is the most life-threatening consequence for cancer patients. In BC, the 5-year survival rate in patients with a primary tumor is 99%, whereas in metastatic set, the 5-year survival rate declined to 23%^20^. This metastatic process comprises many interlacing factors including the cytokines and chemokines levels which have a fundamental role for accomplishing this process^3^. Metastasis of BC is mostly observed in liver, bones, lungs, and brain, but it rarely spreads to other visceral organs^21^. Recently, treatment strategies of metastatic BC are directed for targeting chemokine networks that would have a potential role for improving patients’ outcomes^22^.

The current work demonstrated that IL4, CXCL12, and CCL4 were substantially elevated in BC females in comparison to the control subjects. Whereas there was no significant difference between the BC patients and the control females regarding IL11 and CCL2 concentrations. These data are consistent with many studies reported upregulation of IL4, CXCL12, and CCL4 in BC tissue which were linked with tumor cell proliferation and progression^17,18,23,24^.

Furthermore, IL4 was found to be greatly associated with disease recurrence in BC patients. These data are in agreement with many series reported that type-II IL4 receptors are significantly upregulated in BC, where it promotes BC cells proliferation and metastasis^21,25^. Moreover, it was reported that IL4 has a pro-tumorigenic and immunosuppressive role through polarization of tumor-associated macrophages (TAM) to the M2 phenotype^26^. These TAM-M2 promote cancer progression, invasion, and metastasis of BC cells^27^. Williams et al., demonstrated that increased expression of the type II IL4 receptor associated with reduced overall survival of BC patients through different mechanisms including histone acetylation and activation of Akt/ACLY signaling pathway^28^. Additionally, Rodriguez-Tirado et al., reported that IL4 plays an essential role during seeding and metastasis of BC cells to the lung^29^.

Regarding the CXCL12 expression level, there was a considerable decrease in CXCL12 levels in ER positive BC patients compared to those with ER negative group. Additionally, it was significantly associated with HER2 expression, distant metastasis, poor response to therapy, and disease recurrence in BC patients. These data are in agreement with some previously published articles informed that CXCL12 expression seems to be upregulated in triple negative and HER2 BC subtypes compared with luminal subtype^30,31^. Other studies proposed that high CXCL12 expression was found in BC patients with advanced pathological stage, larger tumor size, positive lymph node metastasis, and poor clinical outcome^32,33^. Similarly, CXCL12 was reported to promote tumor cell proliferation, maintenance of CSCs, resistance to chemotherapy, angiogenesis and metastasis^34,35^.

Moreover, the current study showed that CCL4 increased significantly in patients with positive ER and grade 2 BC tissue. Additionally, there was a notable increase of IL11 in non-TNBC patients, and those with positive ER expression, compared to BC patients with TNBC or low/negative ER expression. These results are comparable to the data found by Franzén et al., that CCL4 concentration associated significantly with higher ER expression in BC tissues^36^. Similarly, Cui et al., reported that increased IL-11Ra expression level was significantly detected in positive ER, positive PR, and low Her-2 BC subtype^37^.

The current results showed also that CCL2 was notably increased in BC patients with distant metastasis and poor response to therapy. These data are similar to that found by Morein et al.,

that CCL2 has an important implication in BC cells progression, invasion, and metastasis^23^. Additionally, recent research supported that CCL2 promotes chemoresistance in BC cells through PI3K/Akt/mTOR signaling pathway activation^38^.

The current study provides evidence that BC bone metastasis associated significantly with increased CCL4 and CCL2 levels, as well as LNs affection. Whereas, multivariate analysis revealed that CCL2 expression, and LNs affection are independent risk factors for bone metastasis in BC patients. In consistence with these findings, Many published articles demonstrated that CCL2 contributed to bone remodeling and osteoclast differentiation to promote bone colonization and metastasis^3,39,40^. Additionally, Palacios-Arreola and his colleagues proposed that CCL2 could enhance BC cells metastasis through inducing CD11b+ monocytes migration resulting in increasing the matrix metalloproteinase-2 (MMP-2), MMP-3, and MMP-9 expression that facilitate tumor cell invasion and metastasis^41^. Moreover, CCL4 was found by many researchers that it is associated significantly with bone metastasis in BC patients^42–46^. This was explained by that CCL4 could activate CCR5 + fibroblasts, which subsequently stimulate connective tissue growth factor (CTGF)/CCN2 within the bone marrow microenvironment, thereby aiding in the growth, survival, and metastasis of BCCs to the bone^42^.

Furthermore, the current work showed that IL11 was significantly increased in BC patients with lung metastasis in relation to those who had no lung metastasis. Univariate analysis also showed that lung metastasis associated significantly with IL11 concentration levels in BC patients. These data are consistent with Tang et al., who found that IL11 exhibited a crucial role for promoting BC cells metastasis and colonization in lung tissue^47^. Therefore, therapeutic targeting of IL-11/IL-11Rα axis could potentially diminish lung metastasis in TNBC patients^47^. Similarly, other studies hypothesized that IL11 could have a pro-tumorogenic role in non-small cell lung cancer (NSCLC) progression as well as extrapulmonary lung metastasis^48,49^. Indeed, the pro-carcinogenic role of IL11 has been explained through various mechanisms including the downstream activation of STAT3 and Akt signaling pathways which results in tumor cells proliferation, invasion, EMT, and metastasis of BC cells to other organs such as liver, lung, and bone^50–52^. Additionally, IL-11/Janus kinase (JAK)/STAT3 activation exerts anti-apoptotic effect through the promotion of Bcl-2 and survivin proteins^48,53^. Consistently, Zhu and his colleagues demonstrated that IL11 produced from lung tissue in response to hypoxia has a critical impact on myofibroblast differentiation through activation of ERK signaling pathway^54^.

Though the clinical outcome of metastatic BC had been improved, about 25% of BC patients experienced brain metastasis. It was reported that BC brain metastasis is the second commonest cause of brain metastasis, which affects the quality of life and survival rates negatively^55^. The current study showed that brain metastasis in BC patients associated significantly with increased IL4 serum level, increased CXCL12 concentration, negative ER, negative PR expression, and increased HER2 expression. Additionally, IL4 and CXCL12 serum levels were substantially elevated in BC patients who had brain metastasis compared to those without brain metastasis. In line with this data, many previous studies reported that brain metastasis is frequently seen in BC patients who had poorly differentiated tumor cells, negative hormone receptors, HER2 positive as well as axillary lymph nodes affection^56–60^. Wang and his team demonstrated in their review that brain metastasis TME has a distinct nature differed from the primary tumor due to the presence of blood brain barrier (BBB), tissue resident cells, as well as the infiltrating immune cells which allow tumor growth and hinder immune rejection^61^. The CTCs secretes many factors including VEGF, cyclooxygenase2, cathepsin S, and CXCL12/CXCR4 that destroy the BBB^62–64^. Accordingly, regulatory cytokines such as IL4 could induce the infiltrating M2 macrophage to produce immune inhibitory molecules including IL10 and TGF-b to allow brain metastasis^65,66^. Similarly, Fearon DT^67^ proposed that CAFs induced immunosuppression of brain metastasis through production of CXCL12 which binds to cancer cells and mediates tumor growth.

Furthermore, the present data showed that low ER expression, brain, and liver metastasis were considered as independent risk factors for shorter DSF rate of the BC patients. These results are in agreement with Cantalejo-Díaz et al. who proposed that liver metastasis and negative hormone receptors are independent prognostic factors for disease recurrence and shorter DFS in BC patients^68^. Quigley et al. also reported that brain metastasis in BC patients is linked to poor prognosis and inferior clinical outcomes of the patients^69^. Additionally, Sun et al., demonstrated that BC with positive HER2 + receptors was more aggressive with increased recurrence rate and shorter overall survival rate of the patients^70^.

In an attempt to link the levels of different circulating cytokines including IL4, IL-11, CCL-2, CCL4 and CXCL-12 with the metastatic site in BC; Roc curve analysis was performed to assess the diagnostic potential of the examined cytokines with the metastatic sites in BC patients. The data revealed that IL4 had a sensitivity of 75% and a specificity of 61.9% (AUC = 0.699) for the association with brain metastasis. Similarly, CXCL12 achieved a sensitivity of 62.5% and a specificity of 81% (AUC = 0.700) for the diagnosis of BC brain metastasis. While the combined expression of both IL4 and CXCL12 exhibited a 75% sensitivity and a 71.4% specificity (AUC = 0.768) for brain metastasis in BC patients. Regarding lung metastasis, IL11 showed a sensitivity of 81.1%, and a specificity of 42.9% (AUC = 0.629) for the diagnosis of BC lung metastasis. Moreover, CCL2 level showed a significant potential for the diagnosis of liver metastasis in BC patients with a sensitivity, specificity, and AUC of 75%, 63.7%, and 0.717; respectively. On the other hand, the plasma levels of IL4, IL11, CCL4, CCL2, and CXCL12 did not show any significant potential for the diagnosis of bone metastasis in BC patients.

In conclusion, IL4, CXCL12, and CCL4 were substantially elevated in BC patients compared to the control group. Low ER expression, presence of brain, and/or liver metastasis were considered as potential independent risk factors for shorter disease-free survival (DFS) of BC patients. Furthermore, the current study provided evidence that chemokines have an important role in directing the CTCs and colonization in a specific metastatic site. According to the data provided in the current work, the significant elevation of the serum levels of IL4 and CXCL12 associated with increased incidence of BC brain metastasis. Meanwhile, the increased circulating level of IL11 accompanied significantly with BC lung metastasis. Bone metastasis is linked with elevated levels of CCL4 and CCL2 in BC patients. Additionally, increased CCL2 concentration is associated with BC liver metastasis. Therefore, these chemokines could be potential therapeutic targets for BC patients to minimize the incidence of metastasis, especially, that these markers achieved a significant diagnostic potential for each metastatic site as previously discussed. Though the study appears to be observational and confirmatory in nature and does not infer causality. The novelty lies in the integrated analysis of multiple cytokines within a single cohort, revealing site-specific cytokine patterns that extend prior single-marker observations and serve as hypothesis-generating data for future mechanistic studies.

The limitations encountered in the current work were that it was designed as cross-sectional to obtain a larger number of samples. Also, there was a relatively small sample size due to multiple subgroupings of the patients according to the metastatic sites, as well as the lack of validation cohort. Therefore, the recommendations were to validate these results on a larger number of patients with different tumor subtypes and grades. Additionally, using a prospective study design with an appropriate validation cohort.

## Data Availability

The datasets used and/or analysed during the current study are available from the corresponding author on reasonable request.
